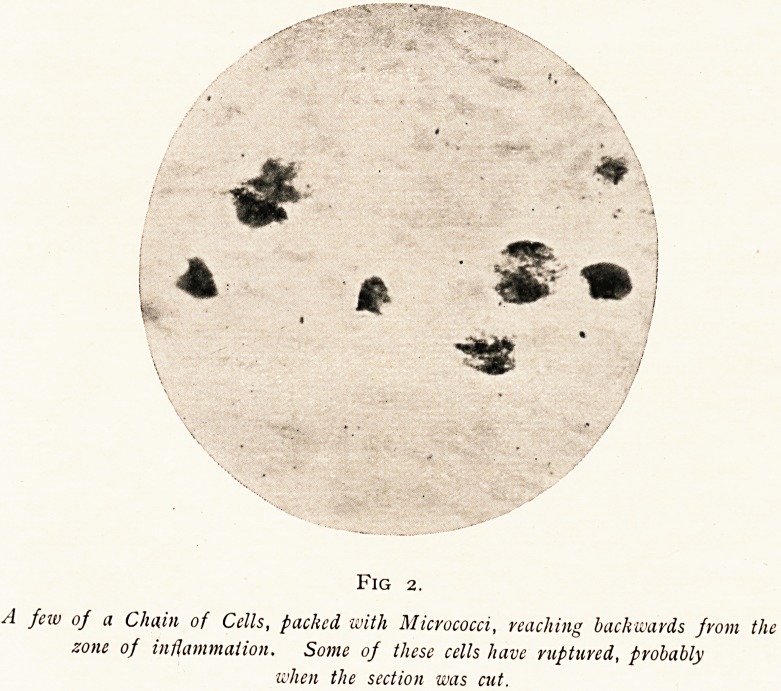# The Origin of Gastric Ulcer

**Published:** 1901-06

**Authors:** W. Gordon

**Affiliations:** Physician to the Royal Devon and Exeter Hospital; Physician to the West of England Eye Infirmary.


### THE ORIGIN OF GASTRIC ULCER.

BY

W. Gordon, M.A., M.D., M.R.C.P.,

*Physician to the Royal Devon and Exeter Hospital; Physician to the West of England Eye Infirmary.*

---

It is a curious fact that, with all our learning, we have learnt so little of the causes of gastric ulcer. One of the commonest of diseases, one of the most important, familiar clinically, only too familiar on the *post-mortem* table, its origin remains an enigma. But we are well off for theories, and these may be stated as follows :—

1. The theory of embolism of a gastric artery.
2. The theory of thrombosis of a gastric artery.
3. The theory of thrombosis of a gastric vein.
4. The theory of spasm of a gastric artery.
5. The theory of hemorrhage into the gastric wall.
6. The theory of injury of the gastric wall from within—
   (*a*) by scalding material;
   (*b*) by corrosive poisons;
   (*c*) by sharp objects swallowed.

7. The theory of injury of the gastric wall from without—
   (*a*) by tumours, aneurysms, &c.;
   (*b*) by mechanical pressure exerted outside the body, as by a tea-tray.
8. The theory of neurotrophic lesion.
9. The theory of autodigestion from hyperacidity.
10. The theory of bacterial necrosis.

And, finally, the theory that all these are true, and what one will not account for another will.

Let us consider these briefly seriatim.

1. *The theory of embolism of a gastric artery.* The idea that somehow or other gastric ulcer is due to some sort of vascular obstruction is that which still dominates medical opinion. When Rokitansky stated that acute perforating gastric ulcers were not due to inflammation, those who accepted his statement had to seek some other explanation. Rokitansky's own explanation that gastric ulcers were due to hemorrhagic erosion met with as little acceptance in his own day as in ours, and the doctrine of vascular obstruction was formulated by Virchow. Virchow seems to have based his arguments first on the asserted absence of inflammation, and secondly on the form and position of gastric ulcers. That embolism of a gastric artery can cause a gastric ulcer is certain, since Panum and Cohnheim have proved it experimentally. But the question is: "Does it cause the disease as we meet with it in practice?"

Emboli may be considered as of two sorts, viz.: (1) masses of microbes circulating in the blood, and (2) vegetations or clots set loose from the heart or vessels. With regard to 1, we plainly have not such to deal with in ordinary cases of gastric ulcer. Microbic emboli have only been met with in cases of anthrax and other infective disorders. It is in respect of 2 that we must carefully consider this theory. If the theory is correct we ought to find that when a source for emboli is evident, gastric ulcer (a common disease, we must remember) should not be rare, and when gastric ulcer is present, we ought not seldom to discover a source for emboli. What are the facts? When many emboli are being thrown off from the heart valves and embolism is appearing in various organs, the gastric arteries almost always escape obstruction. Out of seventy-one cases of ulcerative endocarditis producing emboli in the viscera, recorded by Dr. Fenwick[1] at the London Hospital, not one showed embolism in the stomach. Again, experimentally emboli have been thrown in large numbers into the circulation (Fenwick[2]), yet only 3 to 5 per cent., or less, have found their way into the gastric arteries. The only cases in which this author has seen marked embolism of these arteries were very rare cases of disease (aneurysm) of the neighbouring large vessels.[3] Again, it is a familiar fact that the vast majority of patients who present themselves with gastric ulcers have no disease of either heart or vessels capable of producing emboli.

When we put together these two considerations, the rarity of gastric ulcer where a source of embolism exists, and the rarity of a source of emboli where gastric ulcer exists, can any reasonable person contend that gastric ulcer is the result of embolism ?

2. *The theory of thrombosis of a gastric artery.* Atheroma of the gastric arteries has in some instances been mentioned as a cause of ulcer, and syphilis has been said to have been the cause in others. But it must be obvious that the young women, in whom perforating gastric ulcer is most common, are far below the age at which atheroma occurs, and, in fact, are clinically known to be free from it; whilst syphilis, in country districts at all events, cannot be the cause in the vast majority of patients.

3. *The theory of thrombosis in a gastric vein.* Müller's experiment of ligaturing the portal vein is far too remote from practical bearing to need discussion, and Fenwick's[4] experiments have shown that the ligature of small gastric veins does not lead to ulceration. Thrombi in the veins or arteries of the floor and sides of an ulcer are a part of the effects of ulceration, and prove nothing about its ætiology.

4. *The theory of spasm of a gastric artery.* This has no reasonable evidence in its favour.

5. *The theory of hemorrhage into the gastric walls.* This was

[1] *Ulcer of the Stomach and Duodenum*, 1900, p. 100.

[2] *Op. cit.*, p. 101. [3] *Ibid.* [4] *Op. cit.*, pp. 104–106.

Rokitansky's theory. He recognised that submucous hemorrhage led to erosion, and concluded that ulcer might be caused by hemorrhage. As Pye-Smith points out[1]: "This must be denied, on the ground that if such were its origin it ought to be frequent in those who suffer from obstruction of the portal circulation, as a result of disease of the heart or liver. Hemorrhagic erosions are, in fact, common in cases of this kind, but not gastric ulcer."

6. *The theory of injury of the gastric walls from within.* Scalding material and corrosive poisons can undoubtedly set up ulceration of the stomach, and perhaps sharp objects swallowed may do the same. But no one will contend that the bulk of the cases which come before one clinically are due to any of these causes.

7. *The theory of injury of the gastric walls from without.* Tumours and aneurysms can produce ulceration of the stomach wall, but such cases are curiosities. The pressure-of-tea-tray theory loses sight of the facts that most gastric ulcers are on the posterior wall of the stomach, and that of those which are on the anterior wall the majority are well under cover of the ribs.

8. *The theory of a neurotrophic origin.* This has no evidence worth discussing to support it.

9. *The theory of autodigestion from hyperacidity.* The argument in favour of this theory seems to amount just to this, that "because hyperacidity of the gastric juce is found in about 66 per cent. of the cases of gastric ulcer, therefore the hyperacidity causes the ulcer." So far as I can ascertain, there is no evidence that hyperacidity precedes the ulcer.

10. *The theory of bacterial necrosis.* Bacterial necrosis is a form of "bacterial infection which is unassociated with the signs of active inflammation."[2] At present little seems to be written on this form of microbic injury, but, at all events, no proof has been brought forward of its occurrence in the human stomach, and, as Dreschfeld says, this view may as yet "be looked upon as purely hypothetical."[3]

[1] Fagge and Pye-Smith, *Principles and Practice of Medicine*, 3rd ed., vol ii., 1891, p. 182.

[2] Sidney Martin, *Diseases of the Stomach*, 1895, p. 410.

[3] Dreschfeld, in Clifford Allbutt's *System of Medicine*, vol. iii., 1897, p. 526.

Finally, there is the theory that all these are true. Probably we shall be dealing liberally with the whole of these theories in suggesting that, taken together, they may perhaps account for 5 per cent. of the cases. What, then, is the origin of the remaining 95 per cent.? May we not admit once for all that we know nothing whatever about it? If gastric ulcer were an altogether new disease, and an acute perforating ulcer were shown to anyone who had studied modern pathology, would not inflammation and microbic invasion almost certainly be the first things he would think of? The reason that we, to whom the disease is far from new, do not turn so naturally to these common agencies is, that we have all been brought up so carefully to believe that inflammation has nothing to do with the development of acute gastric ulcer, and that in chronic gastric ulcer the very evident inflammation is not primary but secondary. Yet, is this belief correct? It dates back to a time when the processes of inflammation were less perfectly understood than they are now, and the statements in the text-books read as if they had been copied faithfully one from the other. If acute gastric ulcer is not inflammatory, how does it form adhesions? To argue that the inflammation which forms the adhesions is secondary is merely to beg the question. In very rare instances gastric ulcers have been found to be tubercular. Were these in their origin also non-inflammatory?

Moreover, this belief originated at a time when gastric ulcers were never seen except on the *post-mortem* table, when not merely the ordinary *post-mortem* changes common to all structures, but *post-mortem* digestion as well, had altered the appearances. What might have looked angrily inflamed in life need not necessarily show naked-eye evidence of inflammation after death. Thus the inflammatory appearances described by Beaumont in the living stomach of Alexis St. Martin are quite unknown to us in the pathology of the dead-house. But now in the operating room we have opportunities of seeing gastric ulcers as they exist in life, and we know that gastric ulcers, which have exhibited *post mortem* the characteristic appearances described by Rokitansky, have been seen during life, during operation, as the centre of a considerable apparently inflammatory thickening.[1] It is remarkable in the recent literature of perforating gastric ulcer, how constant is the statement that a considerable area of inflammatory thickening surrounded the perforation. I have collected from the *Lancets* and *British Medical Journals* of the last ten years all the cases I could find of perforated gastric ulcer. In forty-three of these, operation having been performed, the presence or absence of induration was noted at the time. In forty, more or less induration was found to be present, usually very considerable. In only three was it found to be absent. We can scarcely assume that these cases were all, or nearly all, necessarily different from the so-called "acute perforating ulcer;" for the cases were not selected from the journals, and it is unlikely that so few as only three of those operated on were "acute" and all the rest "chronic." They were also mostly on the anterior surface, where "chronic" ulcers are admittedly less common; and whilst one of the three non-indurated had had eighteen months' symptoms of gastric ulcer, one of the forty indurated had had no previous gastric symptoms at all, and two had had symptoms for a few months only. (Many of the reporters gave no previous history of their patients.)

These considerations make one naturally inclined to accept the more recent observations of Jaworski and Korczinski (quoted by Dreschfeld[2]), who find that "the mucous membrane of the stomach in all cases of gastric ulcers shows changes which become more evident on microscopic examination. These changes consist in a cell-infiltration between the several layers of the coats of the stomach and marked inflammatory changes in the walls of the blood-vessels (both of veins and arteries), and in the neighbourhood of the nerves also. According to these authors the inflammatory changes are constantly found in cases of gastric ulcers, and are looked upon by them as primary." In the case of acute ulcer to which I am about to refer, there was indubitable evidence of inflammation round the edge of the aperture. Surely it seems desirable to reconsider the statement that gastric ulcer is non-inflammatory in its origin, and if it should turn out that gastric

[1] Fenwick, *Op. cit.*, p. 19. [2] *Op. cit.*, p. 523.

ulcer is after all an inflammatory disease, to carefully consider whether the inflammation may not be sometimes at least due to micro-organisms. In the case of the rare tubercular ulcerations, it is manifestly due to micro-organisms. Must we not consider the whole question *de novo*?

As matters stand, it may be at least considered permissible to set aside entirely all preconceived ideas of the pathology of gastric ulcer, and to ask ourselves what we might expect to happen if a culture of some common microbe, such as the staphylococcus aureus, were rubbed into the mucous membrane of the stomach.

We are daily swallowing innumerable microbes, and some at least must be supposed to be capable of setting up suppuration in the tissues. These microbes are carried down in an alkaline medium of mixed food and saliva, and in an anæmic servant-girl are apt to be associated with a surplus of saccharine material, in which they have probably already germinated in abundance. They have then presumably twenty minutes or more in the stomach before the alkaline reaction of the food is changed to an acid reaction, and during that time they are being rubbed and scrubbed all over the gastric mucous membrane by the muscular movements of the gastric wall, and kept warm by the warmth of the organ which contains them. We know that a culture of staphylococcus aureus rubbed for a few minutes into the skin of one's arm is very apt to produce inflammation. Should we be greatly astonished if a similar culture scrubbed into the wall of the stomach were to produce inflammation there? If a patch of gastritis or a mechanical erosion existed on the gastric surface, would not such a result be even likelier to follow? Assume for a moment that this is possible, and consider the various consequences which might ensue. The gastric juice fulfils a twofold purpose; it is at once a digesting and a disinfecting fluid. According, therefore, to the virulence of the micro-organisms and the position of their point of entry, we might have one of several results:—

(*a*) If the micro-organisms were virulent, and the point of entry such that the gastric juice did not speedily reach it, it is conceivable that a focus of acute inflammation might develop in the thickness of the gastric wall. That wall is not a very thick one, and it would not require a very long time for serious injury to have been done to a circular area of the size of a threepenny piece, extending through the whole thickness of the coats, but with a rather greater diameter on the side of entry. In most cases this area of acute inflammation would be surrounded by a zone of red and swollen tissue just as a boil is, and, in the centre, necrosis would be tending to begin. Now conceive of the effect on this patch of inflammation of an access of gastric juice. Would not the deeply-injured area be simply digested out, leaving a clean-cut circular "punched-out" opening surrounded with a zone of swollen tissue, more or less infiltrated with leucocytes, close to the margin of solution? Such an ulcer would closely correspond with those now described by operators, and as the swelling round the ulcer would have probably quite subsided after death, (operators have already told us they have found this to be the case), we should have *post-mortem* just the type of lesion with which we are familiar, as "acute perforating ulcer" of the stomach.

(*b*) If, on the other hand, the micro-organisms were less virulent, or their point of entry were such that the tide of gastric juice would reach it before they had penetrated far, no result might follow, or at most a trifling erosion of the surface.

(*c*) Between these extremes all variations should be possible. Sometimes the organisms might have time to effect a superficial lodgment, and to excite a patch of superficial inflammation. Then the gastric juice might digest off their covering, produce a superficial erosion, and destroy them in its floor, leaving one of those surface ulcerations which, on healing, leaves scarcely even puckering behind.

(*d*) Or the organisms might have spread deeper before the gastric juice invaded their superficial layers, and a slow alternation of progress of ulcer and digestion of damaged tissue might proceed indefinitely, together with irritation of the exposed surface, both mechanically by food and chemically by the acid juice. Then we should have steadily-increasing inflammation and a steadily-widening and deepening ulcer, which would adhere to neighbouring organs, and eat its way gradually into their substance. We should, in fact, obtain an ordinary chronic funnel-shaped ulcer.

There is nothing, it seems to me, either inherently improbable or foreign to pathological experience in any of these conceptions. An hypothesis of bacterial origin has, on the contrary, this in its favour, that for a common disease it furnishes a common cause. But we should expect certain other peculiarities to obtain :—

(*a*) We should expect that that part of the stomach where the gastric juice is alkaline at the moment of its secretion would be the part which would be least protected from invasion by micro-organisms, (as Dr. Sidney Martin has pointed out [1]), and therefore we should expect that the pyloric region of the stomach would be a very common situation for a gastric ulcer.

(*b*) We should expect that the part of the stomach which, being most fixed and uppermost, serves to sling the stomach, (in other words, the neighbourhood of the lesser curvature, the part most often out of contact with its acid contents), would suffer more than those parts which are almost continually bathed with acid.

(*c*) We should expect that the parts of this upper portion most remote of all from the gastric juice would be the seat of election of *perforating* ulcers; *i.e.*, that perforating gastric ulcers should be commoner near the cardiac orifice, and, inasmuch as a considerable portion of the twenty-four hours is spent in a recumbent position, they should be also commoner on the front than on the back of the stomach.

(*d*) We should expect, further, that if an open ulcer existed on one wall of the stomach, a second should sometimes form on the opposite wall, where the two walls occasionally came in contact and rubbed on one another.

[1] *Op. cit.*, p. 419.

(*e*) And if one of these two ulcers perforated, we should again expect that, although they would both owe their origin to the same microbe, the anterior ulcer would be the ulcer to give way.

(*f*) We should expect the typical progressive chronic ulcer to be most frequent on the posterior wall of the stomach.

(*g*) We should expect on microscopic section of the edges of an ulcer to find evidence of inflammation, less in the acute, more in the chronic; and certainly we should expect some evidence of microbes in the tissues, microbes growing amongst inflammatory products a little way from the edge, or enclosed in phagocytes in the neighbourhood of the lesion. (Microbes in the floors and edges or sloughs of the ulcers would count for nothing; such might well represent an invasion after the event).

Let us see how far these expected peculiarities are actually found to exist.

(*a*) The pyloric end of the stomach is the commonest seat of gastric ulcer. About 75 per cent. of the ulcers are found there.

(*b*) The neighbourhood of the lesser curvature suffers far more than that of the greater curvature, whilst ulcer of the fundus is very uncommon.

(*c*) Perforating ulcer is much more common on the anterior surface than on the posterior. Brinton found that 70 per cent. of all perforations were on the anterior surface of the stomach. In 111 cases I have collected from the journals (almost all, however, operation cases), in which it is stated that the ulcer was anterior or posterior, 90 or 81 per cent. were on the anterior surface, and 21 or 19 per cent. were on the posterior surface. Again, in 75 of these 111 the position was stated sufficiently clearly to divide the ulcers into those near the cardiac end, those near the pyloric end, and those occupying an intermediate position. Of anterior ulcers, 26 were cardiac and 19 pyloric; and of posterior ulcers 8 were cardiac and 2 pyloric. Thus the neighbourhood of the cardiac orifice and the anterior surface are the commonest seats of the perforating ulcer. Here, however, one must deal in passing with the received explanation of the frequency of anterior perforations. It is said that their greater frequency is due to the greater mobility of the anterior surface, which prevents the formation of adhesions. It seems to me that this reason is unsatisfactory. First, it is by no means certain that the posterior wall of the stomach is so much less mobile than the anterior, for during most of the twenty-four hours the anterior and posterior walls must be equally apt to glide over neighbouring structures, the patient being upright, and most perforations occur when the patient is in an upright position. Secondly, anterior adhesions do form, and not very infrequently. Amongst the 90 cases of *perforating* anterior ulcer, I have found definite mention of adhesions (generally firm adhesions) in 12.

(*d*) The occurrence of two ulcers exactly opposite to each other on the stomach walls is strikingly common. Brinton found amongst 191 cases 24 instances of such opposite ulcers.

(*e*) Sometimes both perforate, but when only one does so, it appears to be always the front one. In Brinton's series there was no exception to this.[1]

(*f*) The typical chronic ulcer is much more common on the posterior than on the anterior wall—as 46 to 7 in Fenwick's recent table.[2]

It will be observed that these peculiarities, which we expect to find on an hypothesis of a microbic origin of gastric ulcer, and actually do find, are the very peculiarities which on other hypotheses have always puzzled us to explain.

(*g*) With regard to the last point, I speak with hesitation. It would be foolish to try to found a theory on a single specimen. But, for what they are worth, I submit some particulars of a perforating gastric ulcer:—Sections of its edge were cut in the expectation of finding evidence of microbic invasion. These sections were stained with

[1] Wilson Fox, *Diseases of the Stomach*, 1872, p. 156. [2] *Op. cit.*, p. 8.

FIG 1.

*Cluster of Micrococci and Scattered Micrococci amongst inflammatory cells, one-eighth of an inch from the edge of the ulcer.*

FIG 2.

*A few of a Chain of Cells, packed with Micrococci, reaching backwards from the zone of inflammation. Some of these cells have ruptured, probably when the section was cut.*

methylene blue, decolourised in slightly acidulated water, dehydrated and mounted in balsam. They showed, close to the digested margin of the perforation, abundant evidence of acute inflammation, abundant migrated leucocytes. About one-eighth of an inch from the margin, just about the middle of the zone of infiltration, clusters of micrococci were found fairly numerous. Some of these are shown in photomicrograph 1. Further from the ulcer, about half an inch from its edge, there was very little leucocytic infiltration, but chains of large cells crammed with cocci could be seen, apparently travelling back from the inflammatory focus along the lymph-paths of the tissues. Some of these are shown in photomicrograph 2. There were no other micro-organisms to be seen within or behind the inflammatory zone. The specimen from which the sections were cut had been some time in spirit, and showed no evidence to the naked eye of inflammatory swelling. One would have chosen it as a typical, acute, punched-out, perforating gastric ulcer. If we assume that these microbes were merely taken up from the raw surface of the ulcer, this difficulty arises, "Why were they all of one sort?"

To my mind the inference seems almost unavoidable, that, in this ulcer, one had to do with the effect of an invasion of the stomach wall by the staphylococcus one found there, and that the process had taken place in the way I have suggested.

At any rate, considering how unsatisfactory are the existing theories, how few of the cases they can collectively account for; how reasonable seems the supposition that gastric ulceration, like other ulcerations, is primarily an inflammatory process; how plausibly, at all events, the peculiarities of the gastric juice will explain the peculiar features of the ulcers, whether acute or chronic; how reasonably we can pass from the supposition that gastric ulcers are inflammatory, to the further supposition that they may be caused by micro-organisms, one cannot but think that even a single case, such as that I have ventured to bring forward, possesses more than a passing interest.

My best thanks are due to my friend, Dr. Norman, for placing at my disposal his incomparable skill as a photo-micrographer. I only regret the imperfections of the specimen which I had to submit to him.

## Figures and Tables

**Fig. 1. f1:**
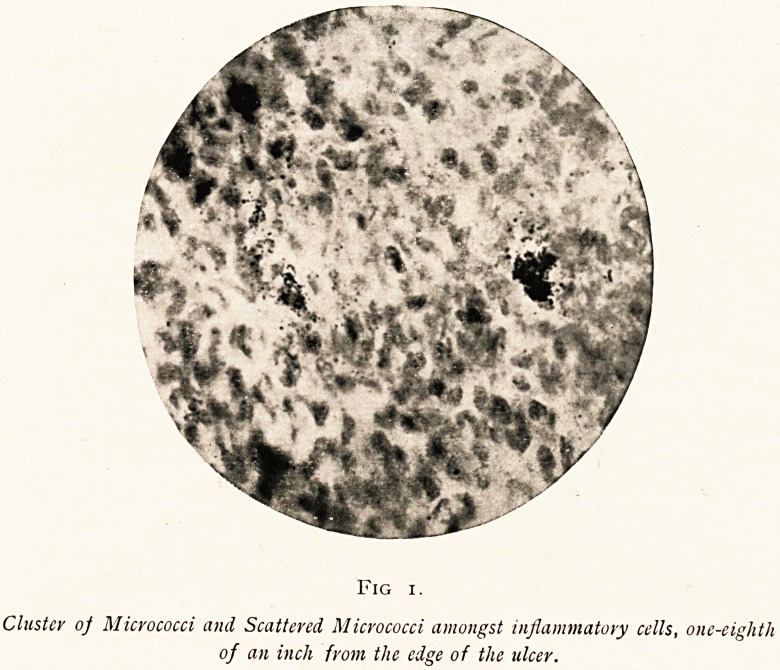


**Fig 2. f2:**